# Validation of the Bronchiectasis Severity Index in alpha-1 antitrypsin deficiency

**DOI:** 10.1371/journal.pone.0355956

**Published:** 2026-09-18

**Authors:** Joshua De Soyza, Paul Ellis, Daniella Spittle, Anita Pye, Alice M. Turner

**Affiliations:** 1 Department of Applied Health Sciences, University of Birmingham, Birmingham, United Kingdom; 2 Department of Respiratory Medicine, University Hospitals Birmingham, Birmingham, United Kingdom; Medizinische Fakultat der RWTH Aachen, GERMANY

## Abstract

Bronchiectasis is increasingly recognised in patients with alpha-1 antitrypsin deficiency (AATD), yet prognostic tools validated in general bronchiectasis populations have not been specifically evaluated in this group. The Bronchiectasis Severity Index (BSI) is a widely used multidimensional score predicting mortality, hospitalisation, and exacerbations in all-cause bronchiectasis. This study aimed to validate the BSI in a cohort of patients with AATD-bronchiectasis. Clinical data were obtained from the Birmingham AATD registry. Patients with severe AATD genotypes and CT-confirmed bronchiectasis were included, while those with non-severe genotypes or alternative causes of bronchiectasis were excluded. BSI scores were calculated using available registry data with minor adjustments reflecting the limitations of cross-sectional data collection. Associations between BSI and mortality were assessed using Kaplan–Meier survival analysis and Cox proportional hazards models adjusted for COPD, smoking status, and sex. Secondary analyses evaluated associations between BSI and lung function decline (FEV_1_ and KCO) and health-related quality of life (SGRQ). A total of 198 patients were included (mean age 54.3 ± 9.7 years; 53.5% male), the majority with the ZZ genotype (97.5%) and coexisting COPD (87.4%). Median BSI score was 5 (range 0–14). Mortality differed significantly across BSI severity groups (p < 0.001): estimated 1-year mortality was 1.1%, 7.4%, and 20.0% for mild, moderate, and severe disease respectively, while 4-year mortality was 5.7%, 23.7%, and 31.8%. In Cox regression analysis adjusted for COPD, smoking, and sex, BSI remained significantly associated with mortality (hazard ratio 1.13 per point increase; 95% CI 1.05–1.23; p = 0.002). BSI score was also associated with greater decline in gas transfer (KCO) (β = −0.22% per point per year; p < 0.001), but showed no significant association with FEV_1_ decline or SGRQ score. The Bronchiectasis Severity Index is associated with mortality risk in patients with AATD-associated bronchiectasis, supporting its use as a prognostic tool in this population. These findings provide evidence that a severity score derived from all-cause bronchiectasis cohorts can be applied to AATD, although prospective validation in larger multicentre datasets is warranted.

## Introduction

Alpha-1 antitrypsin deficiency (AATD) is a rare inherited disorder characterised by low or inactive levels of alpha-1 antitrypsin (AAT), a protease inhibitor. With reduced AAT activity, various proteins, primarily neutrophil elastase and proteinase-3, act unopposed, leading to lung epithelial tissue damage. This tissue damage is typically emphysematous, but AATD has also been linked to bronchiectasis [1,2].

Bronchiectasis is a widely heterogeneous disease defined radiologically as the abnormal dilatation of the bronchi and bronchioles. Disruption of the mucociliary escalator leads to increased mucous production, cough, recurrent chest infections, and shortness of breath, thought to be a result of a vicious cycle consisting of airway inflammation, followed by airway structural damage and dilatation, failure of mucociliary clearance, and susceptibility to bacterial colonisation and infection [3,4]. Bacterial infection causes further airway inflammation, and the cycle continues. All known causes of bronchiectasis fit into this model at one or more stages, and in the case of AATD, unopposed protease activity could instigate the cycle by way of increased airway inflammation. Notably, neutrophil elastase is heavily implicated in the development of idiopathic bronchiectasis [5,6], and in a recent study of the Birmingham AATD cohort, bronchiectasis existed in a significant minority of AATD patients independently of COPD, and was associated with more severe shortness of breath [2].

With readily available multidetector CT scanning a wide range of disease is observed, from incidental diagnoses in asymptomatic patients with mildly dilated bronchi, to cyst-like formations of extremely dilated bronchi in the context of debilitating chronic symptoms and recurrent respiratory infections. Such a broad spectrum has lead to attempts to quantify the disease both radiologically and symptomatically. The Reid classification of morphology is frequently used, although this was originally described based on post-mortem anatomy and predates CT use [7]. Previous studies have also commonly used number of lobes affected as a measure of severity, though some have divided this further into segments affected [8,9]. Many studies have attempted to quantify the degree of airway dilatation, though this is subjective and time-consuming [10,11], and attempts are underway to automate the process to a certain extent [12,13].

With regard to symptoms and prognosis, two scoring systems have been in use over recent years, namely the Bronchiectasis Severity Index (BSI) and the FACED score. The FACED score, originally presented in 2014 [14], includes 5 criteria (Table 1). The FACED score had good predictive power for 5 year all-cause mortality, but did not originally attempt to predict exacerbation rate, a symptom of great importance to patients and healthcare economics, though an extended version was later validated in this regard [15].

**Table 1:** BSI and FACED scores.

Criterion	Points						
	0	1	2	3	4	5	6
***Bronchiectasis Severity Index***							
Age	<50		50-69		70-79		80
BMI	≥18.5		<18.5				
FEV1% predicted	>80	50-80	30-49	<30			
Hospital admission <2 years previously	No					Yes	
Annual exacerbation rate	<3		≥3				
MRC	1-3		4	5			
*Pseudomonas* colonisation	No			Yes			
Colonisation with other pathogens	No	Yes					
≥3 involvement, or cystic morphology	No	Yes					
***FACED***							
FEV1% predicted	≥50		<50				
Age	70		≥70				
Chronic *Pseudomonas* colonisation	No	Yes					
Extension (≥3 lobe involvement)	<3	≥3					
mMRC score [MRC – 1]	0-2	3-4					
*[Extended FACED only]:* *Annual severe exacerbation rate ≥1*	No		Yes				

The BSI of 2014 is more extensive, providing 9 clinical parameters which are independently associated with mortality and hospital admission, and higher scores therefore reflecting poorer prognosis, with one domain for radiological severity, defined as *3 or more lobe involvement or cystic bronchiectasis* [16]. In comparative studies, BSI has been the more capable score in predicting exacerbations, hospitalisation, but with a roughly equal capability to predict mortality [17,18].

AATD is a rare disease, with the ZZ phenotype affecting approximately 250,000 people worldwide, mostly of North and/or Western European ancestry [19]. As such, there is a relative paucity of evidence compared with similar but aetiologically distinct disorders, and therefore information on treatment and prognosis is often extrapolated from these, particularly smoking-related COPD in the case of emphysema, and steatohepatitis and alcohol-related liver disease in the case of liver fibrosis. Patients with the disorder may therefore reasonably have questions about the validity of prognostic information given to them, given that it relies so heavily on information from unrelated disorders, especially in the less common outcomes such as lung transplant. Bronchiectasis is one such outcome, being only observed in a minority of cases of AATD, and even less commonly observed in the absence of concurrent emphysema [2,20].

The aim of this work was therefore to study whether the Bronchiectasis Severity Index, a score validated in all-cause bronchiectasis whose validation cohort did not include AATD, could be validated in a cohort of patients with AATD with bronchiectasis.

## Methods

### Recruitment and data collection

Clinical data was taken from the Birmingham AATD registry, whose prospective data collection process since 1996 has been described previously [21], and includes the constituent parts of the BSI. Patients provide written informed consent to entry into the registry and to the sharing of their data for analysis of disease progression. This process obtained ethical approval in 1996 by the South Birmingham Research and Ethics Committee (Ethics approval number 3359a), and again in 2018 by the South Central – Oxford C Research Ethics Committee (approval number 18/SC/0541). Those attending the tertiary AATD clinic who do not consent are not entered into the database, and consequently are not included in this work; approximately 99% of clinic attendees consent to data entry. All retrospective data was accessed between 01.10.2023 and 01.04.2025. Authors had access to information that could identify individual participants during data collection, as all authors are either clinicians providing NHS care to the same patients, or are otherwise involved in co-ordination and communication with those patients of the research database. Microbiological data was taken from a combination of retrospective data and prospective sample collection. Patients with a history of phlegm production or chronic bronchitis were asked to produce a spontaneous early morning sputum sample and post it to the research laboratory on the same day. Quantitative culture was performed and bacterial isolates were identified. The recruitment period for this study was between 01.10.2023 and 18.5.2024.

Bronchiectasis diagnostic accuracy was checked by reviewing CT scan images, looking for either bronchoarterial ratio >1, lack of airway tapering, or airway visibility within 1 cm of the pleural surface, as per best practice guidelines from the British Thoracic Society. CT images of those with bronchiectasis were assessed for morphology (cylindrical, varicose and cystic) and lobar distribution, which are the CT findings pertinent to the BSI. This assessment was conducted by a physician, with 10% also read by a radiologist. Inter-rater reliability was assessed by the Cohen’s Kappa method.

Patients who had CT scans analysed, but who either did not produce phlegm or who did not send in sputum samples, had their clinical case notes reviewed for any evidence of historic sputum culture results.

### Inclusion and exclusion criteria

The Birmingham AATD registry contains patients with rare variants of AATD whose exact clinical implication is not fully understood, along with several patients with MZ or other “non-severe” phenotypes, who were originally referred by family screening. The statistical analysis was limited to those with severe phenotypes, since these are the patients with clinically relevant AATD, and to optimise applicability to other AATD cohorts. Severe phenotypes were categorised based on literature consensus, with S1 Table giving the full range of phenotypes and categorisation used in our clinic. Those with other causes of bronchiectasis were excluded from statistical analysis.

### Adjustments to BSI scoring

Some adjustments to the BSI parameters were made due to the cross-sectional nature of the data available. The most recent values were taken for body mass index (BMI) and FEV_1_% predicted; median annual exacerbation rate and mode mMRC were used; the age value used was based on age at the last clinic visit, and therefore the age at which most of the other data was taken; and *Pseudomonas* or “other pathogen colonisation” were scored if the patient had *ever* been colonised, as opposed to whether they had been colonised within the last 2 years, as per the original BSI study. Colonisation was defined as 2 or more isolations of the same organism, 3 or more months apart, in line with convention and the original BSI study.

The risk of hospitalisation in the subsequent 2 years, a validated outcome in the original BSI validation study, could not be calculated, as relevant data were not always entered into each patient’s database on the same date, meaning a reference time point for the beginning of the 2-year period could not be identified in many cases.

### Primary and secondary clinical outcomes

The primary outcome was the association of BSI score and mortality.

Secondary outcomes of associations between BSI scores and the secondary clinical outcomes of FEV_1_ and KCO % predicted annual change and mean St George’s Respiratory Questionnaire (SGRQ) score were assessed. These variables were selected in addition to the primary survival analysis, as they are frequently used as indicators of severity of AATD.

### Statistical methods

BSI scores were generated as integers, and also divided into mild (0–4), moderate (5–8), and severe (≥9) categories based on the published method [16], as shown in Table 1. Kaplan-Meier curves were constructed for the association of BSI score with mortality from baseline visit to the AATD clinic in Birmingham, with the log-rank test used to determine statistical significance of differences in survival curves. Cox proportional hazard ratios were also calculated to account for the covariate of COPD, which itself influences mortality, and other variables selected via a backward selection process. The proportional hazards assumption was assessed using Schoenfeld residuals. The presence of COPD was determined by physician-diagnosis, and the accuracy of the diagnosis of COPD was checked by reviewing spirometry for postbronchodilator FEV1/FVC ratio <0.7.

For the secondary outcomes, linear regression models were constructed. Predicted values for FEV_1_ and KCO values were calculated using the Global Lung Initiative formulae [22,23]. Annual change was calculated for those patients who had 3 or more results available. If a linear model identified an association, the clinical variable was compared with BSI severity using standard statistical tests. Model assumptions were evaluated by inspection of residual plots to assess linearity, homoscedasticity, and normality of residuals.

### Additional sensitivity analyses

To determine whether any association with mortality would be primarily reflective of lung function, further sensitivity analyses were conducted. A modified BSI score was constructed, including all BSI variables except FEV_1_% predicted, so that continuous FEV_1_% predicted data could be added into a Cox proportional hazards model as a separate variable. A second Cox proportional hazards model included the full BSI score along with KCO% predicted.

All statistical analysis was performed in RStudio version 2026.0.1.0 using R version 4.5.2.

## Results

### Patient characteristics

**Fig 1:**
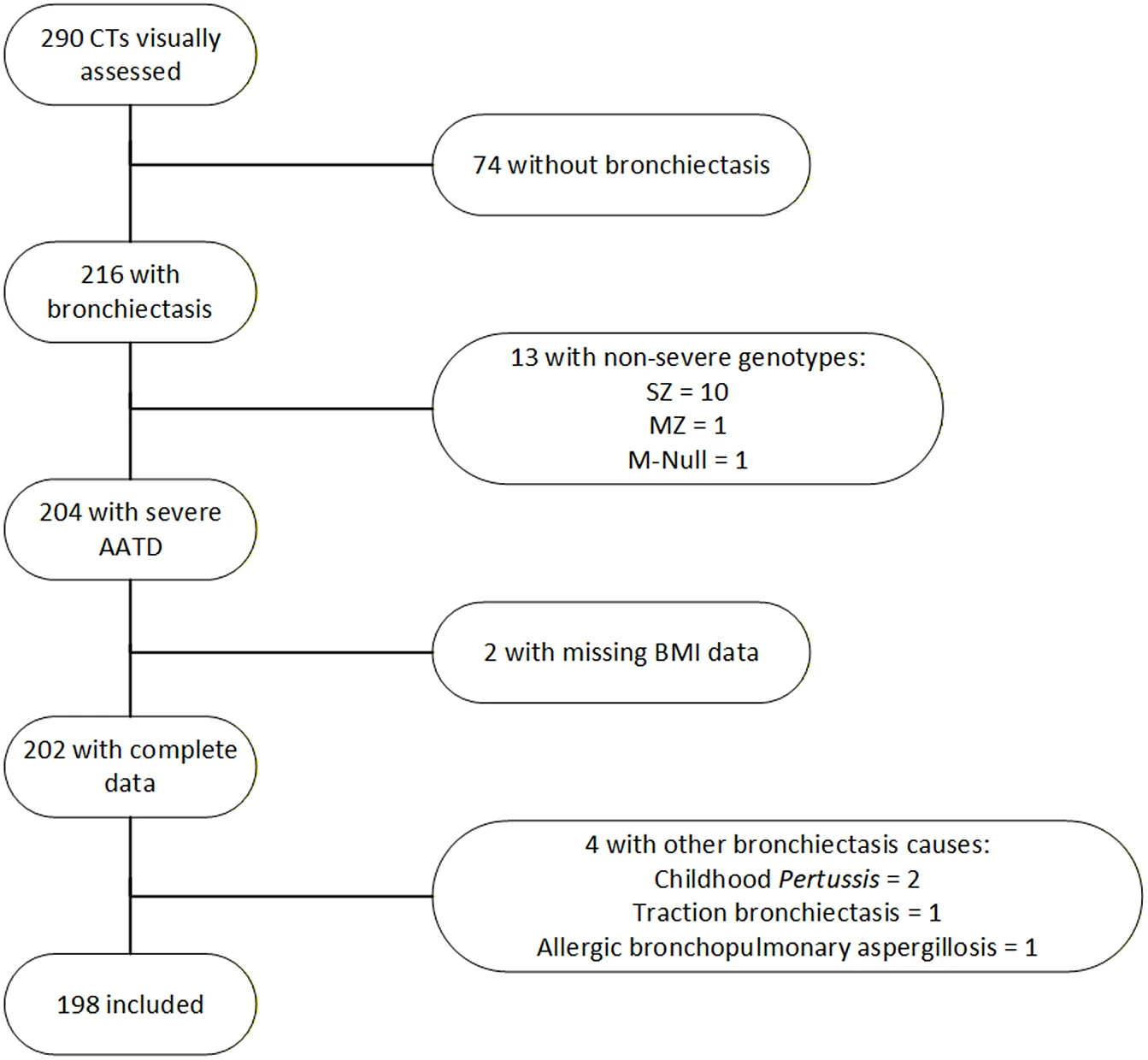
Application of inclusion/exclusion criteria.

The process of inclusion/exclusion of cases is displayed in Fig 1. Of the 290 patients with visually assessed radiology data, 74 did not have bronchiectasis, and were therefore excluded from the calculation of a BSI score. Of the remaining 216 patients, 12 had non-severe genotypes (SZ = 10; M-Null = 1; MZ = 1) and were therefore excluded. 2 further patients were excluded due to missing BMI data which could not be filled. 4 more patients were excluded due to clear alternative causes of bronchiectasis: 2 whose bronchiectasis had been attributed to whooping cough, 1 with traction bronchiectasis secondary to interstitial lung disease, and 1 with allergic-bronchopulmonary aspergillosis. The remaining 198 patients were analysed for correspondence of BSI with mortality; Table 2 gives their baseline characteristics; Table 3 displays their BSI scores for each constituent variable.

**Table 2:** Baseline characteristics of patients included in statistical analysis.

n		198
Age at baseline clinic visit		54.3 (+/- 9.7)
Sex, n (%)	Male	106 (53.5)
Genotype, n (%)	ZZ	193 (97.5)
	FZ	2 (1.0)
	Z-Null	2 (1.0)
	MMalton-Null	1 (0.5)
BMI, mean (SD)		25.3 (+/- 5.5)
Ever-smokers, n (%)		131 (66.2)
COPD, n (%)		173 (87.4)

**Table 3:** Bronchiectasis Severity Index constituent score distributions (n = 198).

Variable		n (%)	BSI score
Age (years, %)	<50	29 (14.6)	0
	50-69	154 (77.8)	2
	70-79	15 (7.6)	4
	≥80	0 (0.0)	6
BMI (%)	≥18.5	191 (96.5)	0
	<18.5	7 (3.5)	2
FEV_1_% predicted (%)	>80	31 (15.7)	0
	50-80	58 (29.3)	1
	30-49	61 (30.8)	2
	<30	48 (24.2)	3
Severe exacerbations (hospitalisations) within last 2 years (%)	None	185 (93.4)	0
	≥1	13 (6.6)	5
Annualised exacerbation rate (%)	<3	175 (88.4)	0
	≥3	23 (11.6)	3
Mode MRC (%)	1-3	121 (61.1)	0
	4	56 (28.3)	2
	5	21 (10.6)	3
Pseudomonas colonisation (%)	No	189 (95.5)	0
	Yes	9 (4.5)	3
Colonisation with other organisms (%)	No	181 (91.4)	0
	Yes	17 (8.6)	1
≥3 lobes involved on CT, or cystic morphology (%)	No	133 (67.2)	0
	Yes	65 (32.8)	1

23 patients provided new sputum samples; these were combined with historic data to give information on *Pseudomonas* and other pathogen colonisation.

### Primary outcomes

**Fig 2:**
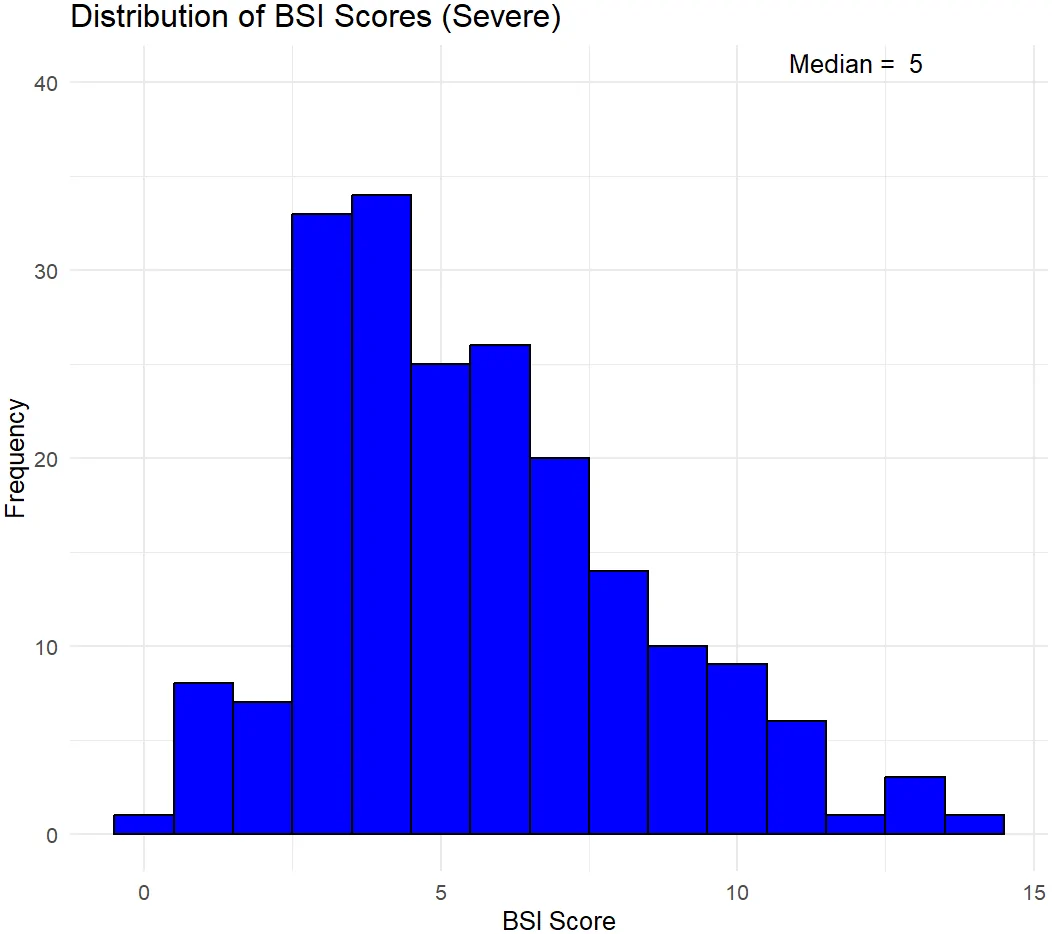
Distribution of BSI scores in AATD patients with severe genotypes.

**Fig 3:**
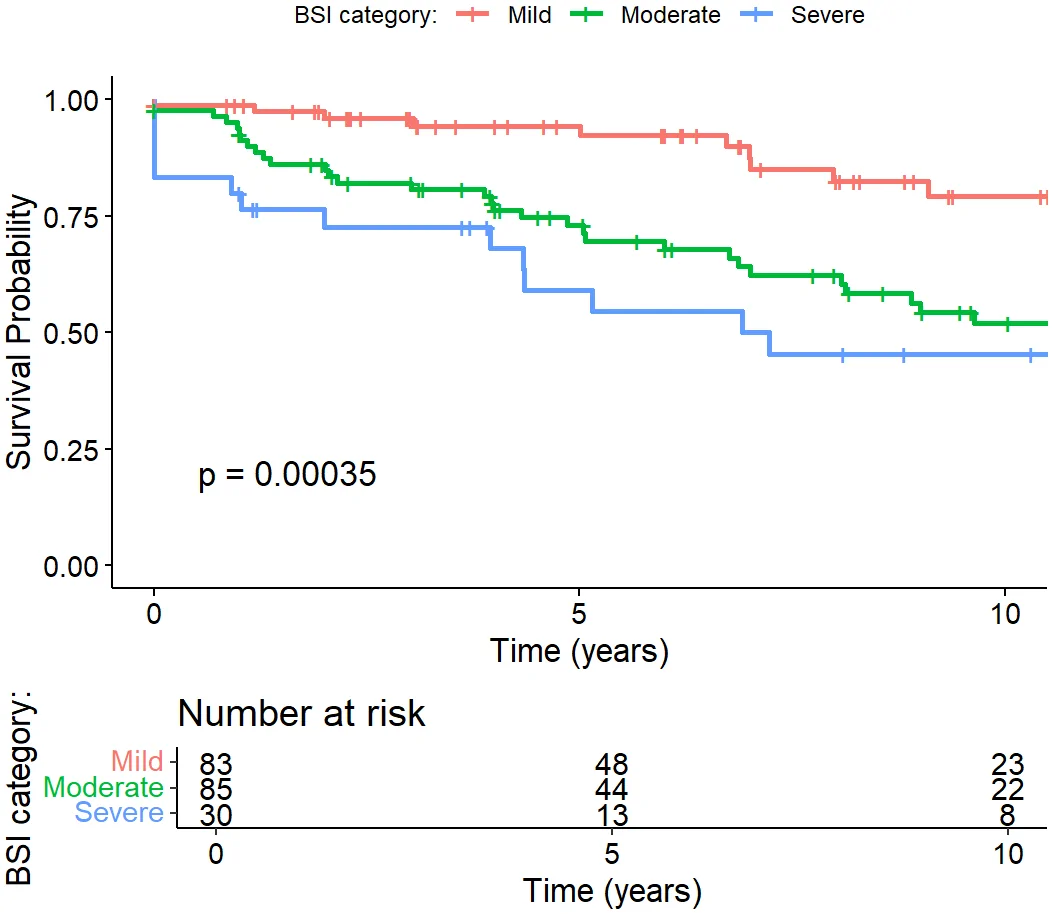
Kaplan-Meier curves comparing BSI severity with mortality from ba*seline.*

Median BSI score was 5, with range 0–14 (Fig 2). Among severe genotypes, survival differed significantly between the 3 groups (Fig 3, p < 0.001). For mild, moderate and severe BSI scores, 1-year mortality was estimated at 1.1, 7.4 and 20.0% respectively, and 4-year mortality was estimated at 5.7, 23.7, and 31.8% respectively. A Cox proportional hazard model correcting for COPD, sex, and smoking status confirmed this difference, with the backward selection model giving a hazard ratio for mortality of 1.13 (95% CI 1.05–1.23, R^2^ = 0.075, p = 0.002).

### Secondary clinical outcomes

There was no evidence of an association between BSI score and annual FEV_1_% predicted change on linear modelling (n = 152, β −0.08, 95% CI −0.18 to 0.02, R^2^ = 0.02, p = 0.12), or between BSI score and mean SGRQ score (n = 81, β 1.37, 95% CI −0.37 to 3.11, R^2^ = 0.03, p = 0.12).

**Fig 4:**
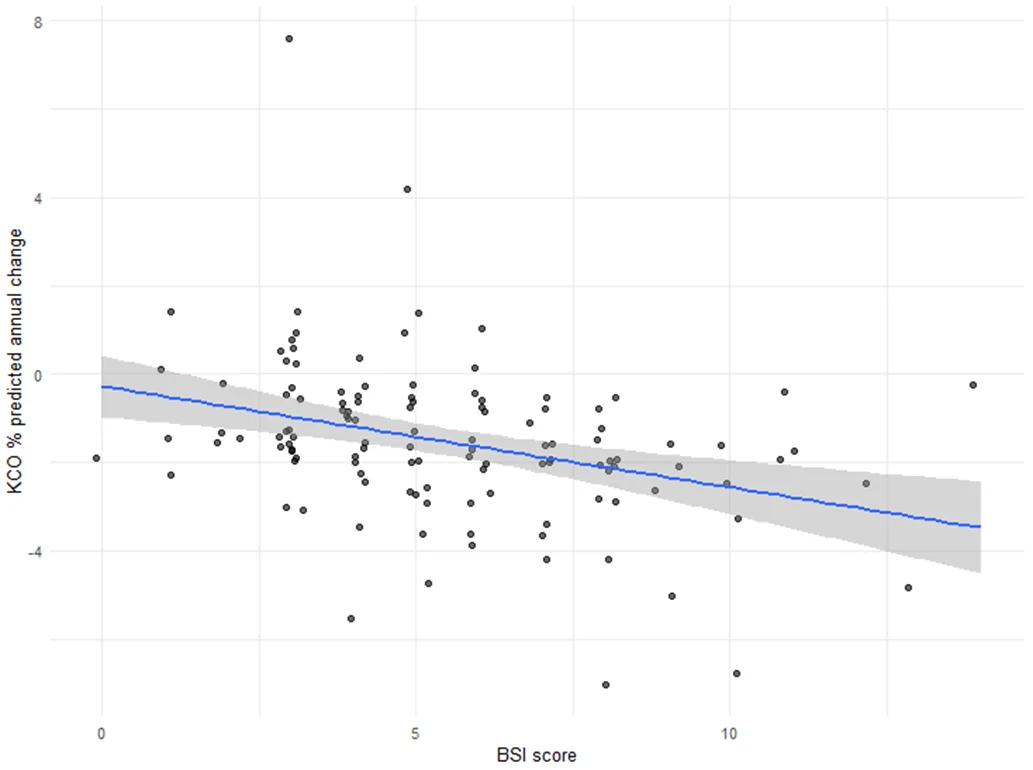
Association between BSI score and KCO % predicted annual change.

However, an association between BSI score and KCO % predicted change was identified (Fig 4), with KCO decline being 0.22% greater with each BSI point (β −0.22, 95% CI −0.34 to −0.11, R^2^ = 0.11, p = 0.00016, n = 121), though 39% (77 of 198) of patients did not have sufficient KCO results to calculate annual change. 11% of variability was explained by the model (R^2^ = 0.11). This association remained statistically significant when the model was adjusted for COPD (β −0.24, 95% CI −0.61 to 1.64, R^2^ = 0.12, p = 0.00012) and when KCO was compared with BSI severity in an ANOVA model (F = 6.59, p = 0.002).

### Additional sensitivity analyses

A Cox proportional hazards model using the modified BSI score along with continuous FEV_1_% predicted data revealed FEV1% predicted to be strongly associated with mortality (HR 0.98, 95% CI 0.97 to 0.99, per 1% predicted increase), though the remaining BSI factors still showed an association with mortality, albeit of reduced statistical significant (HR 1.09, 95% CI 0.99 to 1.19, p = 0.65).

Adjusting the initial Cox proportional hazards model for KCO% predicted revealed both BSI and KCO% predicted to remain associated with mortality (BSI HR 1.15, 95% CI 1.06 to 1.25, p = 0.001; KCO% predicted HR 0.99, 95% CI 0.98 to 1, p = 0.021).

## Discussion

This work presents evidence to support the use of the BSI in AATD, giving patients and their clinicians more confidence in giving accurate estimates of prognosis. This is of great importance, as hitherto both patients and their clinicians may have had doubts in applying this score, due to the differences in underlying aetiology between the original BSI cohort and AATD.

Mortality risk in the three BSI severity groups was broadly similar to those in the BSI derivation study [16]. Although this validation has biological plausibility, some degree of caution in interpretation must be taken, as several parameters had to be adjusted for the cross-sectional nature of this work, whereas BSI was originally validated as a prospective score. However, since a large-scale prospective validation in AATD has been difficult until the recent development of large multi-national AATD databases, this data suffices at present. Prospective work could assess whether BSI may also be predictive of hospital admissions in AATD-bronchiectasis, as the derivation study found [16]. These findings could be compared with matched patients with other causes of bronchiectasis, to confirm whether there are any specific features to AATD-bronchiectasis. Using a single cohort may not result in enough events (admissions) to achieve enough statistical power, so collaborative work with the European Alpha-1 Research Collaboration (EARCO) [24] or similar large databases may be an approach to increase sample size.

BSI score also associated with gas transfer decline, although there was a large amount of missing data and only a modest proportion of variability was explained by the model. Considering anatomy, it is reasonable that more extensive and cystic bronchiectasis should negatively influence diffusing capacity, however these factors are only given a low weighting within the BSI. The variables given greatest weight that are not already accounted for in the GLI calculation of % predicted values are severe exacerbation frequency (i.e., requiring hospitalisation) and *Pseudomonas aeruginosa* colonisation, with radiology, other pathogens, breathlessness and BMI given comparatively smaller weighting (Table 1). *P. aeruginosa* is recognised as a predictor of poorer prognosis in bronchiectasisp [25–27], although a specific association with gas transfer has not been established [27]. Exacerbation frequency has been linked to gas transfer decline in COPD [28], but data in bronchiectasis are lacking. Further studies examining the independent effects of these factors on gas transfer decline in bronchiectasis alone are warranted.

Given most patients in this study had co-existing COPD, mortality predictions for both conditions will have to be interpreted, and the more severe communicated to the patient appropriately. Indeed, in our sensitivity analyses, we found that the major BSI element driving an association in mortality in our patients was FEV_1_% predicted, a marker of severity of COPD. To address this, the BODE index could also be calculated, since it is already frequently used in COPD to predict 4-year survival, and therefore who may benefit from lung transplant [29]. This is less likely to be possible retrospectively, as only a minority of patients in our registry have had formal 6-minute walk tests, a key constituent of the BODE index. Although several parameters of the BODE index and BSI overlap, it would be of great interest to clinicians in AATD, but also in any form of bronchiectasis-COPD overlap syndrome (BCOS), to validate a mortality- and morbidity-predicting score such as BSI or BODE in BCOS, which could lead to the development of a combined score specific to BCOS. Whilst this work has controlled for the presence of COPD in the Cox proportional hazards model, there remains insufficient detail in the literature base to guide clinicians faced with BCOS. In such circumstances, experienced clinicians will rely on their judgement to decide whether to base prognostic estimates on COPD severity (as per GOLD [30] etc) or bronchiectasis severity (as per BSI [16] or FACED [14]). The inclusion of emphysema in the recently developed Bronchiectasis Radiologically Indexed CT Score (BRICS) [31] score can be seen as an acknowledgement of a demand for a severity index combining both diseases. In our cohort it was not possible to include quantitative data on emphysema due to the limitations of the technical variety of scans in the registry, but assessment for any relationship between emphysema and BSI could help to establish the main drivers of mortality in BCOS. This is an important area of future research, and one which should include AATD patients as a cohort in which the two diseases often coexist.

## Conclusion

The most commonly used predictive tool in all-cause bronchiectasis, namely BSI, has been validated in AATD-bronchiectasis as associating with mortality risk from baseline clinic review.

## Supporting information

S1 Table Categorisation of alpha-1 antitrypsin deficiency genotypes (SD = severe deficiency, NS = non-severe, N = normal). (CSV)
