# Supplementary material for: Validation of the Bronchiectasis Severity Index in alpha-1 antitrypsin deficiency
Source: PLoS One. 2026 Sep 18;21(9):e0355956. doi: 10.1371/journal.pone.0355956 (PMC13588374; doi:10.1371/journal.pone.0355956)
Supplement: Supplementary file 1 [file pone.0355956.s003.docx]

Response to reviewers

Journal Requirements:

When submitting your revision, we need you to address these additional requirements.

1. Please ensure that your manuscript meets PLOS ONE's style requirements, including those for file naming. The PLOS ONE style templates can be found at
[https://journals.plos.org/plosone/s/file?id=wjVg/PLOSOne_formatting_sample_main_body.pdf](https://track.editorialmanager.com/CL0/https:%2F%2Fjournals.plos.org%2Fplosone%2Fs%2Ffile%3Fid=wjVg%2FPLOSOne_formatting_sample_main_body.pdf/1/010f019e9ae758f3-22eed128-a224-4633-b559-842d42497056-000000/fibQ22LDDjLGe-UJD-aBue0xu2EupWJiY7t1y30ADEI=258) and <https://journals.plos.org/plosone/s/file?id=ba62/PLOSOne_formatting_sample_title_authors_affiliations.pdf>

*We have done so.*

2. In the ethics statement in the Methods, you have specified that verbal consent was obtained. Please provide additional details regarding how this consent was documented and witnessed, and state whether this was approved by the IRB.

*The manuscript states written consent was obtained in line 103 (rather than verbal). All patients supply written consent before being entered in the Birmingham AATD registry, from which the data for this manuscript is taken. The word “informed” has been added on line 103, which we hope adds extra clarity.*

3. Please include your full ethics statement in the ‘Methods’ section of your manuscript file. In your statement, please include the full name of the IRB or ethics committee who approved or waived your study, as well as whether or not you obtained informed written or verbal consent. If consent was waived for your study, please include this information in your statement as well.

*The recruitment process for the AATD registry from which our data was taken obtained ethical approval in 1996 by the South Birmingham Research and Ethics Committee (Ethics approval number 3359a), and again in 2018 by the South Central - Oxford C Research Ethics Committee (approval number 18/SC/0541). The manuscript has been updated from line 104 to reflect this extra detail.*

4. Thank you for stating the following in the Competing Interests section:
I have read the journal's policy and the authors of this manuscript have the following competing interests:

PE has received speaker fees from Chiesi, GSK, and AstraZeneca. AMT has had grants to her institution and/or honoraria from CSL Behring, Grifols, Vertex, Takeda, Chiesi, AstraZeneca, GSK, Sanofi, and Boehringer Ingelheim. JDS, DA, and AP have no conflicts of interest to declare.

Please confirm that this does not alter your adherence to all PLOS ONE policies on sharing data and materials, by including the following statement: "This does not alter our adherence to PLOS ONE policies on sharing data and materials.” (as detailed online in our guide for authors <http://journals.plos.org/plosone/s/competing-interests>). If there are restrictions on sharing of data and/or materials, please state these. Please note that we cannot proceed with consideration of your article until this information has been declared.

Please include your updated Competing Interests statement in your cover letter; we will change the online submission form on your behalf.

We confirm that the competing interests do not alter our adherence to PLOS ONE policies on sharing data and materials. We will include this information in the revised cover letter.

5. Please amend your authorship list in your manuscript file to include author Joshua De Soyza, Paul Ellis, Daniella Spittle, Anita Pye, Alice M Turner.

We have done so

6. Please amend the manuscript submission data (via Edit Submission) to include author J De Soyza1, P Ellis, D Spittle, A Pye, AM Turner.

We have done so

7. Please include captions for your Supporting Information files at the end of your manuscript, and update any in-text citations to match accordingly. Please see our Supporting Information guidelines for more information: http://journals.plos.org/plosone/s/supporting-information.

 We have done so

8. If the reviewer comments include a recommendation to cite specific previously published works, please review and evaluate these publications to determine whether they are relevant and should be cited. There is no requirement to cite these works unless the editor has indicated otherwise. 


[Note: HTML markup is below. Please do not edit.]

Reviewers' comments:

Reviewer's Responses to Questions

**Comments to the Author**

5. Review Comments to the Author

Reviewer #1: 
Comment and Suggestion for Improvement
Additional clarification regarding the characterization of bronchiectasis (e.g., radiological criteria and differentiation from COPD-dominant phenotypes) would further strengthen the manuscript.

Additional clarification has been added in the paragraph starting at line 126: “Bronchiectasis accuracy was checked by reviewing CT scan images, looking for either bronchoarterial ratio >1, lack of airway tapering, or airway visibility within 1cm of the pleural surface, as per best practice guidelines from the British Thoracic Society. CT images of those with bronchiectasis were assessed for morphology (cylindrical, varicose and cystic) and lobar distribution, which are the CT findings pertinent to the BSI. This assessment was conducted by a physician, with 10% also read by a radiologist. Inter-rater reliability was assessed by the Cohen’s Kappa method.”

Reviewer #2: The manuscript addresses an important and clinically relevant question, namely the prognostic value of the BSI in patients with AATD-associated bronchiectasis. The study is well motivated, as bronchiectasis is increasingly recognized in AATD and prognostic tools validated in general bronchiectasis populations have not been specifically evaluated in this setting. The analyses appear robust and the findings are of potential clinical interest.

I have several comments that may further strengthen the manuscript:

1.) Definition of COPD: The authors adjusted their survival analyses for the presence of COPD. However, the diagnosis of COPD in patients with bronchiectasis can be challenging, where airflow obstruction may arise from multiple overlapping mechanisms. It would therefore be helpful if the authors could clarify how COPD was defined in this cohort. Was COPD based on physician diagnosis, spirometric criteria, or both? If COPD was defined clinically, I would encourage the authors to report how many of these patients fulfilled spirometric criteria for airflow obstruction . This information would improve the interpretability of the adjustment model.

This has been clarified in lines 178-181 – COPD was defined as a physician diagnosis, but each case was reviewed for post-bronchodilator FEV1/FVC ratio <0.7.

2.) Independence of the BSI from lung function impairment.

Since FEV₁ contributes directly to the BSI calculation and airflow obstruction is a major determinant of prognosis in AATD, it would be informative to better understand the main drivers of the observed association between BSI and mortality. In particular, it remains unclear whether the prognostic signal of the BSI primarily reflects bronchiectasis severity itself or underlying impairment in lung function. I would therefore encourage the authors to perform an additional sensitivity analysis including FEV₁ % predicted as a continuous variable in the Cox proportional hazards model.

Furthermore, given the well-established prognostic value of impaired gas transfer in AATD, it would be highly informative to assess whether the association between BSI and mortality remains significant after adjustment for DLCO (or KCO) % predicted. Such analyses could help determine whether the BSI provides prognostic information beyond established markers of pulmonary impairment and may offer insights into the principal factors driving the observed increase in mortality risk.

BSI already includes FEV1 percent predicted, stratified into 4 levels, and therefore adding FEV1% into the CPH model would introduce a high degree of collinearity. However, we ran a Cox model which included FEV1% predicted, and a modified BSI score with the FEV1 score removed. This model showed FEV1% predicted to be strongly associated with mortality (HR 0.98, 95% CI 0.97 to 0.99, per 1% predicted increase), though the remaining BSI factors still showed an association with mortality, albeit of reduced statistical significant (HR 1.09, 95% CI 0.99 to 1.19, p = 0.65).

A Cox proportional hazards model was constructed to include BSI and KCO% predicted, with results showing both BSI and KCO% predicted to remain associated with mortality (BSI HR 1.15, 95% CI 1.06 to 1.25, p = 0.001; KCO% predicted HR 0.99, 95% CI 0.98 to 1, p = 0.021).

These methods and results have been added to the manuscript at lines 191 and 251 onwards, respectively, with a small discussion line added at line 298.

3.) Role of emphysema Given the central role of emphysema in AATD, it would be interesting to know whether CT-derived data on emphysema extent or severity were available within the cohort. If such data exist, exploratory analyses examining the relationship between emphysema burden, BSI, and mortality could provide valuable additional insights. A

We thank the reviewer for this excellent point. Some quantitative emphysema data is available within our cohort, however this is only for a minority, and the missing data is such that the model would drop the majority of patients, leaving only 94 of the original 198. We have therefore elected not to continue with this analysis, although this would be an interesting area of future research in our group as quantitative methods of analysis become easier to access prospectively, as we have mentioned in our discussion from line 316.

Overall, this is a valuable study addressing an important gap in the literature. Clarification of the COPD definition and additional analyses exploring the independence of BSI from lung function parameters and the potential contribution of emphysema would further strengthen the manuscript.
