# Supplementary material for: Validation of the Bronchiectasis Severity Index in alpha-1 antitrypsin deficiency
Source: PLoS One. 2026 Sep 18;21(9):e0355956. doi: 10.1371/journal.pone.0355956 (PMC13588374; doi:10.1371/journal.pone.0355956)
Supplement: Peer review history

# Peer review: Decision Letter 0

**DOI:** 10.1371/journal.pone.0355956.r001

**Authors:** Pavel Strnad

5 Jun 2026

Dear Dr. De Soyza,

Please submit your revised manuscript by Jul 20 2026 11:59PM. If you will need more time than this to complete your revisions, please reply to this message or contact the journal office at plosone@plos.org. When you're ready to submit your revision, log on to https://www.editorialmanager.com/pone/ and select the 'Submissions Needing Revision' folder to locate your manuscript file.

- A letter that responds to each point raised by the academic editor and reviewer(s). You should upload this letter as a separate file labeled 'Response to Reviewers'.
- A marked-up copy of your manuscript that highlights changes made to the original version. You should upload this as a separate file labeled 'Revised Manuscript with Track Changes'.
- An unmarked version of your revised paper without tracked changes. You should upload this as a separate file labeled 'Manuscript'.

If you would like to make changes to your financial disclosure, please include your updated statement in your cover letter. Guidelines for resubmitting your figure files are available below the reviewer comments at the end of this letter.

If applicable, we recommend that you deposit your laboratory protocols in protocols.io to enhance the reproducibility of your results. Protocols.io assigns your protocol its own identifier (DOI) so that it can be cited independently in the future. For instructions see: https://journals.plos.org/plosone/s/submission-guidelines#loc-laboratory-protocols. Additionally, PLOS ONE offers an option for publishing peer-reviewed Lab Protocol articles, which describe protocols hosted on protocols.io. Read more information on sharing protocols at https://plos.org/protocols?utm_medium=editorial-email&utm_source=authorletters&utm_campaign=protocols.

As the corresponding author, your ORCID iD is verified in the submission system and will appear in the published article. PLOS supports the use of ORCID, and we encourage all coauthors to register for an ORCID iD and use it as well. Please encourage your coauthors to verify their ORCID iD within the submission system before final acceptance, as unverified ORCID iDs will not appear in the published article. *Only*  the individual author can complete the verification step; PLOS staff *cannot*  verify ORCID iDs on behalf of authors.

We look forward to receiving your revised manuscript.

Kind regards,

Pavel Strnad

Academic Editor

PLOS One

Journal Requirements:

When submitting your revision, we need you to address these additional requirements.

1. Please ensure that your manuscript meets PLOS ONE's style requirements, including those for file naming. The PLOS ONE style templates can be found at

https://journals.plos.org/plosone/s/file?id=wjVg/PLOSOne_formatting_sample_main_body.pdf and https://journals.plos.org/plosone/s/file?id=ba62/PLOSOne_formatting_sample_title_authors_affiliations.pdf

2. In the ethics statement in the Methods, you have specified that verbal consent was obtained. Please provide additional details regarding how this consent was documented and witnessed, and state whether this was approved by the IRB.

3. Please include your full ethics statement in the ‘Methods’ section of your manuscript file. In your statement, please include the full name of the IRB or ethics committee who approved or waived your study, as well as whether or not you obtained informed written or verbal consent. If consent was waived for your study, please include this information in your statement as well.

4. Thank you for stating the following in the Competing Interests section:

I have read the journal's policy and the authors of this manuscript have the following competing interests:

PE has received speaker fees from Chiesi, GSK, and AstraZeneca. AMT has had grants to her institution and/or honoraria from CSL Behring, Grifols, Vertex, Takeda, Chiesi, AstraZeneca, GSK, Sanofi, and Boehringer Ingelheim. JDS, DA, and AP have no conflicts of interest to declare.

Please confirm that this does not alter your adherence to all PLOS ONE policies on sharing data and materials, by including the following statement: "This does not alter our adherence to PLOS ONE policies on sharing data and materials.” (as detailed online in our guide for authors http://journals.plos.org/plosone/s/competing-interests). If there are restrictions on sharing of data and/or materials, please state these. Please note that we cannot proceed with consideration of your article until this information has been declared.

Please include your updated Competing Interests statement in your cover letter; we will change the online submission form on your behalf.

5. Please amend your authorship list in your manuscript file to include author Joshua De Soyza, Paul Ellis, Daniella Spittle, Anita Pye, Alice M Turner.

6. Please amend the manuscript submission data (via Edit Submission) to include author J De Soyza1, P Ellis, D Spittle, A Pye, AM Turner.

7. Please include captions for your Supporting Information files at the end of your manuscript, and update any in-text citations to match accordingly. Please see our Supporting Information guidelines for more information: http://journals.plos.org/plosone/s/supporting-information.

8. If the reviewer comments include a recommendation to cite specific previously published works, please review and evaluate these publications to determine whether they are relevant and should be cited. There is no requirement to cite these works unless the editor has indicated otherwise.

[Note: HTML markup is below. Please do not edit.]

Reviewers' comments:

Reviewer's Responses to Questions

**Comments to the Author**

1. Is the manuscript technically sound, and do the data support the conclusions?

Reviewer #1: Yes

Reviewer #2: Yes

**********

2. Has the statistical analysis been performed appropriately and rigorously?

Reviewer #1: Yes

Reviewer #2: Yes

**********

3. Have the authors made all data underlying the findings in their manuscript fully available?

The PLOS Data policy

Reviewer #1: Yes

Reviewer #2: Yes

**********

4. Is the manuscript presented in an intelligible fashion and written in standard English?

Reviewer #1: Yes

Reviewer #2: Yes

**********

Reviewer #1: This manuscript presents an evaluation of the Bronchiectasis Severity Index (BSI) in a well-characterized cohort of patients with alpha-1 antitrypsin deficiency (AATD), focusing on its association with mortality. The topic is clinically relevant, as bronchiectasis is increasingly recognized in AATD, yet validated prognostic tools in this specific population remain limited.

General Assessment

Overall, this is a well-conducted and clearly presented study. The use of a relatively large, single-center cohort with detailed clinical characterization strengthens the analysis. The finding that BSI is associated with mortality in AATD-associated bronchiectasis is important and may have practical implications for risk stratification in this population.

Strengths

The study addresses a clinically meaningful and underexplored question.

The cohort appears well-defined, with genetically confirmed severe AATD cases.

The use of an established and widely used severity index (BSI) enhances clinical applicability.

The demonstration of an association between BSI and mortality provides useful prognostic insight.

Comment and Suggestion for Improvement

Additional clarification regarding the characterization of bronchiectasis (e.g., radiological criteria and differentiation from COPD-dominant phenotypes) would further strengthen the manuscript.

Reviewer #2: The manuscript addresses an important and clinically relevant question, namely the prognostic value of the BSI in patients with AATD-associated bronchiectasis. The study is well motivated, as bronchiectasis is increasingly recognized in AATD and prognostic tools validated in general bronchiectasis populations have not been specifically evaluated in this setting. The analyses appear robust and the findings are of potential clinical interest.

I have several comments that may further strengthen the manuscript:

1.) Definition of COPD: The authors adjusted their survival analyses for the presence of COPD. However, the diagnosis of COPD in patients with bronchiectasis can be challenging, where airflow obstruction may arise from multiple overlapping mechanisms. It would therefore be helpful if the authors could clarify how COPD was defined in this cohort. Was COPD based on physician diagnosis, spirometric criteria, or both? If COPD was defined clinically, I would encourage the authors to report how many of these patients fulfilled spirometric criteria for airflow obstruction . This information would improve the interpretability of the adjustment model.

2.) Independence of the BSI from lung function impairment.

Since FEV₁ contributes directly to the BSI calculation and airflow obstruction is a major determinant of prognosis in AATD, it would be informative to better understand the main drivers of the observed association between BSI and mortality. In particular, it remains unclear whether the prognostic signal of the BSI primarily reflects bronchiectasis severity itself or underlying impairment in lung function. I would therefore encourage the authors to perform an additional sensitivity analysis including FEV₁ % predicted as a continuous variable in the Cox proportional hazards model.

Furthermore, given the well-established prognostic value of impaired gas transfer in AATD, it would be highly informative to assess whether the association between BSI and mortality remains significant after adjustment for DLCO (or KCO) % predicted. Such analyses could help determine whether the BSI provides prognostic information beyond established markers of pulmonary impairment and may offer insights into the principal factors driving the observed increase in mortality risk.

3.) Role of emphysema Given the central role of emphysema in AATD, it would be interesting to know whether CT-derived data on emphysema extent or severity were available within the cohort. If such data exist, exploratory analyses examining the relationship between emphysema burden, BSI, and mortality could provide valuable additional insights. A

Overall, this is a valuable study addressing an important gap in the literature. Clarification of the COPD definition and additional analyses exploring the independence of BSI from lung function parameters and the potential contribution of emphysema would further strengthen the manuscript.

**********

what does this mean?). If published, this will include your full peer review and any attached files.

If you choose “no”, your identity will remain anonymous but your review may still be made public.

**Do you want your identity to be public for this peer review?**  For information about this choice, including consent withdrawal, please see our Privacy Policy

Reviewer #1: No

Reviewer #2: No

**********

[NOTE: If reviewer comments were submitted as an attachment file, they will be attached to this email and accessible via the submission site. Please log into your account, locate the manuscript record, and check for the action link "View Attachments". If this link does not appear, there are no attachment files.]

To ensure your figures meet our technical requirements, please review our figure guidelines: https://journals.plos.org/plosone/s/figures

You may also use PLOS’s free figure tool, NAAS, to help you prepare publication quality figures: https://journals.plos.org/plosone/s/figures#loc-tools-for-figure-preparation.

NAAS will assess whether your figures meet our technical requirements by comparing each figure against our figure specifications.

# Author comment: Author response to Decision Letter 1

**DOI:** 10.1371/journal.pone.0355956.r002

13 Jul 2026

Response to reviewers

Journal Requirements:

When submitting your revision, we need you to address these additional requirements.

1. Please ensure that your manuscript meets PLOS ONE's style requirements, including those for file naming. The PLOS ONE style templates can be found at

https://journals.plos.org/plosone/s/file?id=wjVg/PLOSOne_formatting_sample_main_body.pdf and https://journals.plos.org/plosone/s/file?id=ba62/PLOSOne_formatting_sample_title_authors_affiliations.pdf

We have done so.

2. In the ethics statement in the Methods, you have specified that verbal consent was obtained. Please provide additional details regarding how this consent was documented and witnessed, and state whether this was approved by the IRB.

The manuscript states written consent was obtained in line 103 (rather than verbal). All patients supply written consent before being entered in the Birmingham AATD registry, from which the data for this manuscript is taken. The word “informed” has been added on line 103, which we hope adds extra clarity.

3. Please include your full ethics statement in the ‘Methods’ section of your manuscript file. In your statement, please include the full name of the IRB or ethics committee who approved or waived your study, as well as whether or not you obtained informed written or verbal consent. If consent was waived for your study, please include this information in your statement as well.

The recruitment process for the AATD registry from which our data was taken obtained ethical approval in 1996 by the South Birmingham Research and Ethics Committee (Ethics approval number 3359a), and again in 2018 by the South Central - Oxford C Research Ethics Committee (approval number 18/SC/0541). The manuscript has been updated from line 104 to reflect this extra detail.

4. Thank you for stating the following in the Competing Interests section:

I have read the journal's policy and the authors of this manuscript have the following competing interests:

PE has received speaker fees from Chiesi, GSK, and AstraZeneca. AMT has had grants to her institution and/or honoraria from CSL Behring, Grifols, Vertex, Takeda, Chiesi, AstraZeneca, GSK, Sanofi, and Boehringer Ingelheim. JDS, DA, and AP have no conflicts of interest to declare.

Please confirm that this does not alter your adherence to all PLOS ONE policies on sharing data and materials, by including the following statement: "This does not alter our adherence to PLOS ONE policies on sharing data and materials.” (as detailed online in our guide for authors http://journals.plos.org/plosone/s/competing-interests). If there are restrictions on sharing of data and/or materials, please state these. Please note that we cannot proceed with consideration of your article until this information has been declared.

Please include your updated Competing Interests statement in your cover letter; we will change the online submission form on your behalf.

We confirm that the competing interests do not alter our adherence to PLOS ONE policies on sharing data and materials. We will include this information in the revised cover letter.

5. Please amend your authorship list in your manuscript file to include author Joshua De Soyza, Paul Ellis, Daniella Spittle, Anita Pye, Alice M Turner.

We have done so

6. Please amend the manuscript submission data (via Edit Submission) to include author J De Soyza1, P Ellis, D Spittle, A Pye, AM Turner.

We have done so

7. Please include captions for your Supporting Information files at the end of your manuscript, and update any in-text citations to match accordingly. Please see our Supporting Information guidelines for more information: http://journals.plos.org/plosone/s/supporting-information.

We have done so

8. If the reviewer comments include a recommendation to cite specific previously published works, please review and evaluate these publications to determine whether they are relevant and should be cited. There is no requirement to cite these works unless the editor has indicated otherwise.

[Note: HTML markup is below. Please do not edit.]

Reviewers' comments:

Reviewer's Responses to Questions

Comments to the Author

5. Review Comments to the Author

Reviewer #1:

Comment and Suggestion for Improvement

Additional clarification regarding the characterization of bronchiectasis (e.g., radiological criteria and differentiation from COPD-dominant phenotypes) would further strengthen the manuscript.

Additional clarification has been added in the paragraph starting at line 126: “Bronchiectasis accuracy was checked by reviewing CT scan images, looking for either bronchoarterial ratio >1, lack of airway tapering, or airway visibility within 1cm of the pleural surface, as per best practice guidelines from the British Thoracic Society. CT images of those with bronchiectasis were assessed for morphology (cylindrical, varicose and cystic) and lobar distribution, which are the CT findings pertinent to the BSI. This assessment was conducted by a physician, with 10% also read by a radiologist. Inter-rater reliability was assessed by the Cohen’s Kappa method.”

Reviewer #2: The manuscript addresses an important and clinically relevant question, namely the prognostic value of the BSI in patients with AATD-associated bronchiectasis. The study is well motivated, as bronchiectasis is increasingly recognized in AATD and prognostic tools validated in general bronchiectasis populations have not been specifically evaluated in this setting. The analyses appear robust and the findings are of potential clinical interest.

I have several comments that may further strengthen the manuscript:

1.) Definition of COPD: The authors adjusted their survival analyses for the presence of COPD. However, the diagnosis of COPD in patients with bronchiectasis can be challenging, where airflow obstruction may arise from multiple overlapping mechanisms. It would therefore be helpful if the authors could clarify how COPD was defined in this cohort. Was COPD based on physician diagnosis, spirometric criteria, or both? If COPD was defined clinically, I would encourage the authors to report how many of these patients fulfilled spirometric criteria for airflow obstruction . This information would improve the interpretability of the adjustment model.

This has been clarified in lines 178-181 – COPD was defined as a physician diagnosis, but each case was reviewed for post-bronchodilator FEV1/FVC ratio <0.7.

2.) Independence of the BSI from lung function impairment.

Since FEV₁ contributes directly to the BSI calculation and airflow obstruction is a major determinant of prognosis in AATD, it would be informative to better understand the main drivers of the observed association between BSI and mortality. In particular, it remains unclear whether the prognostic signal of the BSI primarily reflects bronchiectasis severity itself or underlying impairment in lung function. I would therefore encourage the authors to perform an additional sensitivity analysis including FEV₁ % predicted as a continuous variable in the Cox proportional hazards model.

Furthermore, given the well-established prognostic value of impaired gas transfer in AATD, it would be highly informative to assess whether the association between BSI and mortality remains significant after adjustment for DLCO (or KCO) % predicted. Such analyses could help determine whether the BSI provides prognostic information beyond established markers of pulmonary impairment and may offer insights into the principal factors driving the observed increase in mortality risk.

BSI already includes FEV1 percent predicted, stratified into 4 levels, and therefore adding FEV1% into the CPH model would introduce a high degree of collinearity. However, we ran a Cox model which included FEV1% predicted, and a modified BSI score with the FEV1 score removed. This model showed FEV1% predicted to be strongly associated with mortality (HR 0.98, 95% CI 0.97 to 0.99, per 1% predicted increase), though the remaining BSI factors still showed an association with mortality, albeit of reduced statistical significant (HR 1.09, 95% CI 0.99 to 1.19, p = 0.65).

A Cox proportional hazards model was constructed to include BSI and KCO% predicted, with results showing both BSI and KCO% predicted to remain associated with mortality (BSI HR 1.15, 95% CI 1.06 to 1.25, p = 0.001; KCO% predicted HR 0.99, 95% CI 0.98 to 1, p = 0.021).

These methods and results have been added to the manuscript at lines 191 and 251 onwards, respectively, with a small discussion line added at line 298.

3.) Role of emphysema Given the central role of emphysema in AATD, it would be interesting to know whether CT-derived data on emphysema extent or severity were available within the cohort. If such data exist, exploratory analyses examining the relationship between emphysema burden, BSI, and mortality could provide valuable additional insights. A

We thank the reviewer for this excellent point. Some quantitative emphysema data is available within our cohort, however this is only for a minority, and the missing data is such that the model would drop the majority of patients, leaving only 94 of the original 198. We have therefore elected not to continue with this analysis, although this would be an interesting area of future research in our group as quantitative methods of analysis become easier to access prospectively, as we have mentioned in our discussion from line 316.

Attachment Submitted filename: BSI AATD Response to reviewers.docx

# Peer review: Decision Letter 1

**DOI:** 10.1371/journal.pone.0355956.r003

**Authors:** Pavel Strnad

28 Jul 2026

Validation of the Bronchiectasis Severity Index in Alpha-1 Antitrypsin Deficiency

PONE-D-26-15459R1

Dear Dr. De Soyza,

We’re pleased to inform you that your manuscript has been judged scientifically suitable for publication and will be formally accepted for publication once it meets all outstanding technical requirements.

Within one week, you’ll receive an e-mail detailing the required amendments. When these have been addressed, you’ll receive a formal acceptance letter and your manuscript will be scheduled for publication.

An invoice will be generated when your article is formally accepted. Please note, if your institution has a publishing partnership with PLOS and your article meets the relevant criteria, all or part of your publication costs will be covered. Please make sure your user information is up-to-date by logging into Editorial Manager at Editorial Manager® and clicking the ‘Update My Information' link at the top of the page. For questions related to billing, please contact billing support.

If your institution or institutions have a press office, please notify them about your upcoming paper to help maximize its impact. If they’ll be preparing press materials, please inform our press team as soon as possible -- no later than 48 hours after receiving the formal acceptance. Your manuscript will remain under strict press embargo until 2 pm Eastern Time on the date of publication. For more information, please contact onepress@plos.org.

Kind regards,

Pavel Strnad

Academic Editor

PLOS One

Additional Editor Comments (optional): Congratulations to the nice work!

Reviewers' comments:

Reviewer's Responses to Questions

**Comments to the Author**

Reviewer #1: All comments have been addressed

**********

2. Is the manuscript technically sound, and do the data support the conclusions?

Reviewer #1: Yes

**********

3. Has the statistical analysis been performed appropriately and rigorously?

Reviewer #1: Yes

**********

4. Have the authors made all data underlying the findings in their manuscript fully available?

The PLOS Data policy

Reviewer #1: Yes

**********

5. Is the manuscript presented in an intelligible fashion and written in standard English?

Reviewer #1: Yes

**********

Reviewer #1: Thank you for the opportunity to review the revised manuscript entitled “Validation of the Bronchiectasis Severity Index in Alpha-1 Antitrypsin Deficiency.” The authors have addressed the previous comments thoroughly, and the manuscript has improved significantly in clarity, methodological transparency, and overall scientific quality. The study addresses a clinically relevant gap and provides valuable evidence supporting the use of the Bronchiectasis Severity Index in patients with AATD. I have no more recommendations.

**********

what does this mean?). If published, this will include your full peer review and any attached files.

If you choose “no”, your identity will remain anonymous but your review may still be made public.

**Do you want your identity to be public for this peer review?**  For information about this choice, including consent withdrawal, please see our Privacy Policy

Reviewer #1: No

**********

# Editor report: Acceptance letter

**DOI:** 10.1371/journal.pone.0355956.r004

**Authors:** Pavel Strnad

PONE-D-26-15459R1

PLOS One

Dear Dr. De Soyza,

I'm pleased to inform you that your manuscript has been deemed suitable for publication in PLOS One. Congratulations! Your manuscript is now being handed over to our production team.

At this stage, our production department will prepare your paper for publication. This includes ensuring the following:

* All references, tables, and figures are properly cited

* All relevant supporting information is included in the manuscript submission,

* There are no issues that prevent the paper from being properly typeset

You will receive further instructions from the production team, including instructions on how to review your proof when it is ready. Please keep in mind that we are working through a large volume of accepted articles, so please give us a few days to review your paper and let you know the next and final steps.

Lastly, if your institution or institutions have a press office, please let them know about your upcoming paper now to help maximize its impact. If they'll be preparing press materials, please inform our press team within the next 48 hours. Your manuscript will remain under strict press embargo until 2 pm Eastern Time on the date of publication. For more information, please contact onepress@plos.org.

You will receive an invoice from PLOS for your publication fee after your manuscript has reached the completed accept phase. If you receive an email requesting payment before acceptance or for any other service, this may be a phishing scheme. Learn how to identify phishing emails and protect your accounts at https://explore.plos.org/phishing.

If we can help with anything else, please email us at customercare@plos.org.

Thank you for submitting your work to PLOS One and supporting open access.

Kind regards,

PLOS One Editorial Office Staff

on behalf of

Dr. Pavel Strnad

Academic Editor

PLOS One
